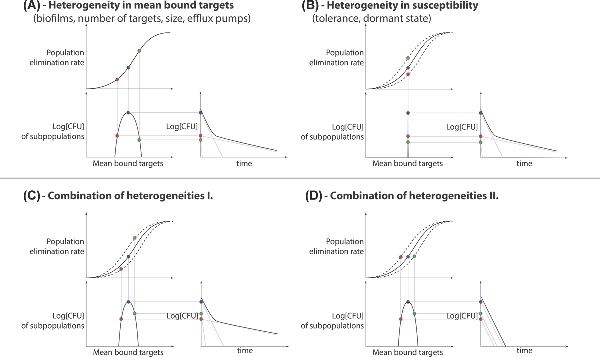# Estimating treatment prolongation for persistent infections

**DOI:** 10.1093/femspd/fty065

**Published:** 2018-08-09

**Authors:** Antal Martinecz, Pia Abel zur Wiesch

**Affiliations:** 1 Department of Pharmacy, Faculty of Health Sciences, UiT The Arctic University of Norway, 9037 Tromsø; 2 Centre for Molecular Medicine Norway, Nordic EMBL Partnership, P.O. Box 1137, Blindern, 0318 Oslo, Norway

**Keywords:** persistence, antimicrobial, treatment length, mathematical model, bacteria, antibiotic

## Abstract

Treatment of infectious diseases is often long and requires patients to take drugs even after they have seemingly recovered. This is because of a phenomenon called persistence, which allows small fractions of the bacterial population to survive treatment despite being genetically susceptible. The surviving subpopulation is often below detection limit and therefore is empirically inaccessible but can cause treatment failure when treatment is terminated prematurely. Mathematical models could aid in predicting bacterial survival and thereby determine sufficient treatment length. However, the mechanisms of persistence are hotly debated, necessitating the development of multiple mechanistic models. Here we develop a generalized mathematical framework that can accommodate various persistence mechanisms from measurable heterogeneities in pathogen populations. It allows the estimation of the relative increase in treatment length necessary to eradicate persisters compared to the majority population. To simplify and generalize, we separate the model into two parts: the distribution of the molecular mechanism of persistence in the bacterial population (e.g. number of efflux pumps or target molecules, growth rates) and the elimination rate of single bacteria as a function of that phenotype. Thereby, we obtain an estimate of the required treatment length for each phenotypic subpopulation depending on its size and susceptibility.

## INTRODUCTION

**Figure 1.**
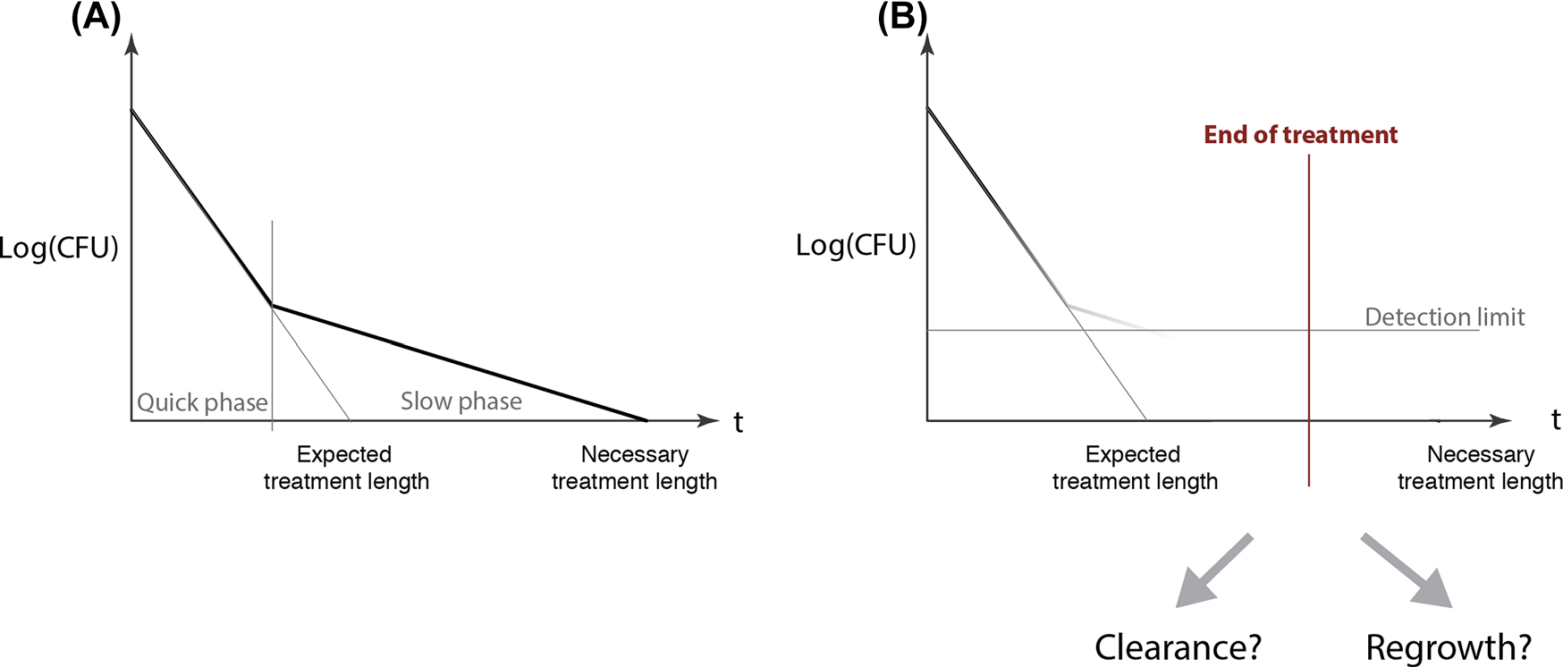
Persistence lengthens treatment. **A** and **B**, Shows the change in the number of bacteria (colony forming units, CFU) over time. The curve is a typical biphasic kill-curve associated with persistence. In these cases, a fraction of the population may be below the detection limit. Therefore, if the treatment is terminated at the expected treatment length based on only initial elimination rates; a fraction on the population might survive. As these bacteria are still viable, they might regrow and cause relapse. As the Y-axis is logarithmic, exponential decays are straight lines that cross the X-axis at one individual bacterium. We assume that at this time point the population is eliminated. **B**, Illustrates how persisters below the detection limit can complicate treatments by making it difficult to assess whether it is safe to end a treatment.

Antibacterial treatments can be lengthy for certain diseases. An extreme example is tuberculosis, where the treatment lasts between 6 and 24 months (Lawn and Zumla 2011; Horsburgh, Barry and Lange 2015). As the treatment length increases, patient adherence can dramatically drop (Burnier *et al.*^2013^), which in turn can increase the treatment length even further while also inflating its costs. A major barrier in reducing treatment length is the risk of relapse: if bacterial populations are below detection limit but not eliminated, bacteria may regrow resulting in treatment failure (Fig. 1). This can be due to various different mechanisms, for example bacteria hiding in ‘sanctuary sites’ (Claudi *et al.*^2014^; Kaiser *et al.*^2014^), in cells (Prajsnar *et al.*^2012^), granulomas (Monack, Bouley and Falkow 2004; Lawn and Zumla 2011) or the phenomenon of persistence in a single bacterial population (Balaban *et al.*^2004^; Lewis 2007; Ankomah and Levin 2014; Abel zur Wiesch *et al.*^2015^; Bergmiller *et al.*^2017^). In this work we focus on the latter, specifically in bacterial populations.

Persistent bacteria survive prolonged exposure to antibiotics, despite being genetically susceptible. This phenomenon is often characterized by bi- or multi-phasic kill-curves: after the initial rapid elimination of bacteria, the rate of elimination slows down. Therefore, as depicted in Fig. 1, bacteria can survive for a longer time than one would expect based on the initial elimination rates.

The nature and cause of bacterial persistence during antibiotic therapy is hotly debated (Balaban *et al.*^2013^). Antibiotic persistence models can be divided into two (nonexclusive) groups. First, the ‘classical’ models that assume a distinct phenotype or state that causes persistence. Here, the minority of persister cells and the majority of normal cells are clearly distinct (Balaban *et al.*^2004^; Lewis 2007) and antibiotic susceptibility in the bacterial population follows a bimodal distribution (in the extreme case no antibiotic susceptibility for persisters and full susceptibility for ‘normal’ cells). Second, ‘heterogeneity’ based models that assume a (unimodal) distribution in bacterial susceptibility. Here, persisting bacteria are not distinct, but are at the tail of the susceptibility distribution (Wakamoto *et al.*^2013^; Fridman *et al.*^2014^; Abel zur Wiesch *et al.*^2015^). We have previously demonstrated that minor heterogeneities in drug-target binding are sufficient to explain multiphasic kill-curves and thus persistence as we currently understand it (Abel zur Wiesch *et al.*^2015^). Moreover, our work made the prediction that a larger variance in molecular heterogeneity increases persister-like behavior that has now been confirmed experimentally (Rego, Audette and Rubin 2017).

The purpose of this work is to estimate the impact of persistence on the time required until all bacteria are cleared by antibiotics. Specifically, we develop a generalized framework that can be used on various mechanisms of persistence, including persistence against antimicrobial peptides or other agents of the host's immune system. While the principles can be applied to all types of distributions of persister phenotypes, we focus on bacterial persistence due to heterogeneity in susceptibility that follows a Gaussian distribution. Having a common framework allows the comparison of various mechanisms, as well as applying them in the same model, and consequently investigating their combined effects on treatment length. While persistence against antibiotics in bacteria has received most attention, there is also evidence that elimination by the immune system can also result in bi- or multi-phasic kill-curves. For example, the dynamics of antibodies binding to the epitopes of HIV virions (Magnus and Regoes 2011; Magnus *et al.*^2013^), cell-to-cell variability in the apoptosis of cancer cells (Spencer *et al.*^2009^), persisters’ tolerance towards serum-complement mediated killing (Putrinš *et al.*^2015^), or local adaptation to-, and heterogeneity in the immune clearance of bacterial colonies (Bumann 2015).

Here, we present a mathematical modeling framework that allows translating population heterogeneity in antibiotic susceptibility (uni-, bi- or multi-modal) into time–kill curves and the required treatment time to eradicate a bacterial population. This modeling framework consist of two parts: first, a simplified model that allows translating drug target binding in homogeneous bacterial subpopulations into elimination rates. The simplified model is applicable to any mechanism of pathogen killing where step-wise binding or molecular recognition is involved. Second, estimating prolongation of treatment length in bacterial populations with heterogeneities in elimination rates. This part of the modeling framework is independent of the mechanism responsible for the heterogeneity in elimination rates and can be used to infer the heterogeneities’ effects on treatment prolongation. We also show this with an experimental dataset as an example on how to use our methodology.

## MATERIALS AND METHODS

In the following, we first describe the response of a single bacterium to successive binding of a killing agent and then the response of a bacterial population (elimination rate) to an increasing number of mean bound target molecules. Finally, we show how heterogenous elimination rates of individual subpopulations can be used to estimate the increase in treatment length caused by persisters.

### Inferring bacterial elimination rates from drug-target binding

#### Response of single bacteria to antibiotic binding

Whether bacteria are killed by antibiotics, the immune system or even viruses by antivirals, molecular binding and recognition is arguably involved in all cases. Therefore, models that incorporate subsequent binding events are useful for understanding the response to drugs or the immune system (Magnus and Regoes 2011; Shen *et al.*^2011^; Magnus *et al.*^2013^). Previously, we have demonstrated (Abel zur Wiesch *et al.*^2015^), that bacterial populations with heterogeneity in the number of targets can show persistent behavior. The first aim here is to generalize and simplify this model.

**Figure 2.**
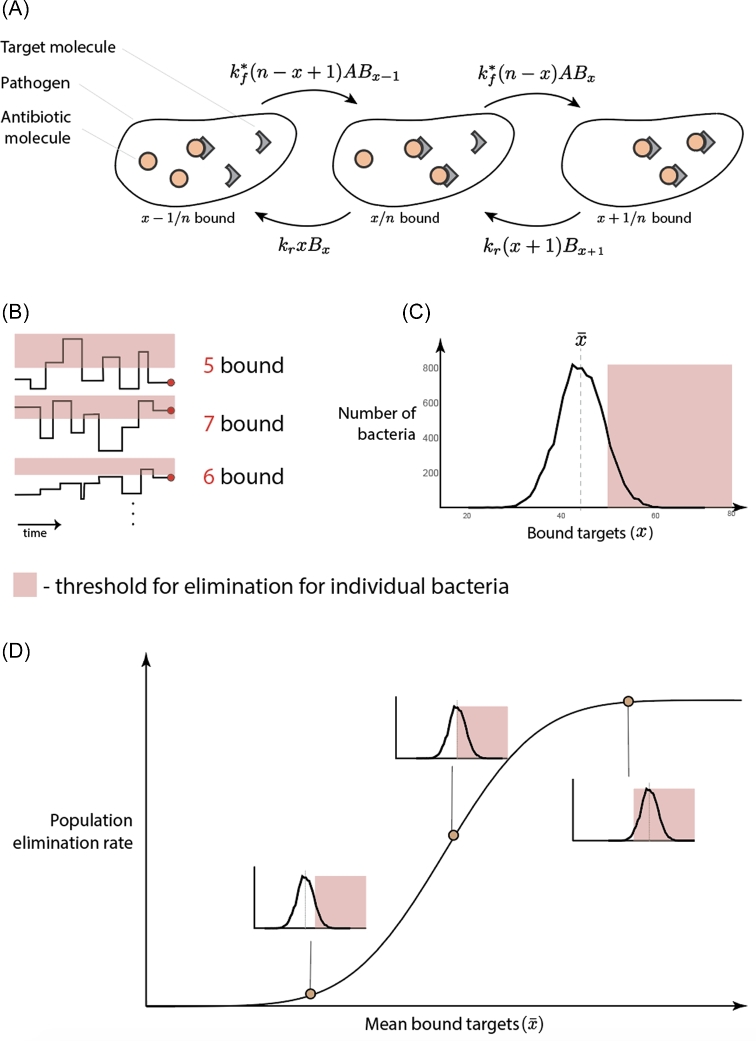
Overview of the mathematical model describing successive binding steps. **A**, Illustrates how the binding kinetics are calculated (Equation 1). Here, $k_f^*$ is the adjusted forward reaction rate (see below), *k_r_* is the reverse reaction rate, *A* is the concentration of antibiotics inside the bacterium, *x* is the number of bound targets, *n* is the number of targets and *B_x_* is the number of bacteria with *x* bound targets. **B**, Shows how the random binding and unbinding of targets causes a variance in the number of bound targets around the mean and how this causes the individual bacterium to cross the number of bound targets required for elimination. **C**, Gillespie simulation of Equation (1). This plot shows the distribution around the mean. The parameters used are *k_d_* = 10 ^− 5^ (ciprofloxacin, (Spratt 1975; Terrak *et al.*^1999^)), number of targets *n* = 100 (gyrase, (Malmström *et al.*^2009^; Maier *et al.*^2011^)). **D**, Shows how the random binding and unbinding (Figure B and C) will cause a fraction of population to die earlier than expected that results in a net elimination rate. The curve on Figure D was obtained using Equation (4).

In (Abel zur Wiesch *et al.*^2015^), we set up a mathematical model, where we calculated the following for multiple subpopulations with different number of targets (Fig. 2, and Equation 1): first, number of antibiotic molecules inside each cell based on external antibiotic concentrations. Second, the number of bacteria with *x* bound targets (within each subpopulation), and finally the replication and elimination for bacteria with *x* bound targets (for each *x* in each subpopulation). In that model, we assumed that the antibiotic concentration is the same inside and outside the cells. Therefore, the number of antibiotic molecules inside each bacterium can be estimated based on the average volume of a bacterium and the external concentration.

Our model describes successive binding steps, meaning that each compartment/differential equation (*B_x_*) contains and keeps track of the number of bacteria with the given number of bound targets (*x*) (see Fig. 2).

The differential equations of the binding kinetics are:

(1) \begin{eqnarray*} \frac{{d{B_x}}}{{dt}} &=& k_f^*\ A\left( t \right){B_{x - 1}}\left( {n - \left( {x - 1} \right)} \right) - {k_r}x\ {B_x}\nonumber\\ && -\, k_f^*A\left( t \right){B_x}\left( {n - x} \right) + {k_r}\left( {x + 1} \right){B_{x + 1}}, \end{eqnarray*}

where:

- $k_f^* = \frac{{{k_f}}}{{{V_i} \cdot {n_A}}}$ is the adjusted forward reaction rate to accommodate working with the number molecules inside the cells instead of concentrations. Here, *k_f_* is the forward reaction rate, *V_i_* = 10 ^− 15^ [*l*] is the average cell volume, and *n_A_* = 6 · 10^23^ is the Avogadro number,
- *k_r_*is the reverse reaction rate,
- *A*(t) is the concentration of antibiotics in or around the bacterium,
- *x*is the number of bound targets,
- *n*is the number of targets,
- *B_x_*is the number of bacteria with *x* bound targets.

All the models mentioned above (Magnus and Regoes 2010; Shen *et al.*^2011^; Abel zur Wiesch *et al.*^2015^) assume for simplicity that the elimination rate function for a single bacterium is a step function. Once a threshold in the number of bound targets is reached, the bacterium dies or, in the case of virions, becomes noninfectious.

The model gets significantly simpler if it is sufficient to calculate the mean number of bound targets instead of calculating the full reaction kinetics model (Equation 1). This can be done if the binding kinetics and bacterial replication/elimination rates can be separated from each other. The condition for this is that the binding rates have to be at least a magnitude faster than the change of external antibiotic concentrations or the replication/elimination rates. In other words, they have to be on different time-scales. In these cases, the reaction kinetics will always reach steady state before any new replication/elimination ‘event’ happens, and the changes in antibiotic concentration is also closely followed by the reaction kinetics due to the differences in the timescales the two are acting on. Consequently, when calculating the binding kinetics, the two other processes can be regarded as ‘constant’. This is the case for slow growing bacteria (e.g. *Mycobacterium tuberculosis*) and when antibiotic concentrations are well above the minimum inhibitory concentration (MIC), as we have shown in (Abel zur Wiesch, Clarelli and Cohen 2017). For other cases, this depends on the binding rates and concentrations of the antibiotics, as well as the replication of rates of the given bacterium. As we assume that there is no change in the number of target molecules through bacterial death, our model would also be applicable in cases where dead bacteria or at least their target molecules are not degraded and continue participating in drug binding.

With this simplification, the time course of the mean number of bound targets ($\bar{x}$) can be determined by using classical reaction kinetics ($A + T \mathbin{\lower.3ex\hbox{$\buildrel\textstyle\leftharpoonup\over {\smash{\rightharpoondown}}$}} AT$, Equation 2) between the free targets ($n - \ \bar{x}$) and the antibiotics (*A*):

(2) \begin{eqnarray*} \frac{{d\bar{x}}}{{dt}} = {k_f} A\left( t \right)\left( {n - \bar{x}} \right)\ - {k_r}\bar{x} \end{eqnarray*}

If A(t) fluctuates slowly compared to the settling time of the reaction, the equilibrium of this time course is at:

(3) \begin{eqnarray*} \nu = n\frac{{A\left( t \right)}}{{\frac{{kr}}{{kf}} + A\left( t \right)}} \end{eqnarray*}

After separation of reaction kinetics from the replication and elimination processes, the binding kinetics reduced to a problem of calculating the number of bound targets at a constant antibiotic concentration. Therefore, the number of mean bound targets can be determined using Equations (2) or (3) and the distribution of bound targets around it (as depicted on Fig. 2C) can be approximated with a Gaussian distribution (van Kampen 2007).

In the following, we will investigate how a population of bacteria responds to an increasing number of bound targets, which in turn is dependent on the antibiotic concentration (Equations 2 and 3).

#### *Response of bacterial population to successive drug binding (i.e. dose-response curves)*

On a population-level, we expect a step function in single cell response to result in a sigmoidal elimination curve: the random binding and unbinding of antibiotics and their targets creates a dynamic equilibrium around the mean number of bound targets ($\bar{x}$). Consequently, the number of bound targets will occasionally reach the threshold for elimination long before the mean number of bound target reaches it (see Fig. 2). The frequency with which bacteria reach that threshold by chance and are therefore killed increases with the antibiotic concentration and this results in a sigmoidal curve, as has been observed in experimental data (Regoes *et al.*^2004^).

Based on the distribution around the mean bound targets, it is possible to estimate an effective elimination rate curve for a homogeneous population of bacteria (see Fig. 2D, and Equation 4). As a result, the only input necessary for the effective elimination rate curve is now the mean number of bound targets.

(4) \begin{eqnarray*} {\delta ^*} \left({\bar{x}} \right) = \mathop \sum \limits_x \delta \left( x \right)B\left( {x,\ \ \bar{x}} \right), \end{eqnarray*}

where:

- $B( {x,\ \ \bar{x}})$ is the distribution of bound targets around the mean $( {\bar{x}} )$ (Fig. 2B and C)
- δ(*x*) is the elimination rate depending on the number of bound targets (Fig. 2B and C)
- ${\delta ^*}( {\bar{x}} )$ is the effective, population-wide elimination rate, only depends on the mean bound targets (Fig. 2D).

### Integrating population heterogeneity on dose-response curves

Bacterial populations often contain various heterogeneities that can cause heterogeneities in elimination rates, for example the numbers of efflux pumps (Pu *et al.*^2016^; Bergmiller *et al.*^2017^), or sizes of bacteria and therefore the intracellular target concentrations (Abel zur Wiesch *et al.*^2015^; Rego, Audette and Rubin 2017) can vary from cell to cell.

In (Abel zur Wiesch *et al.*^2015^) among others, we have demonstrated that the effects of small heterogeneities in the number of targets can lead to biphasic time–kill curves. Here, heterogeneity in the number of targets can be included the following way: the relationship between the number of targets (*n*) and mean bound targets ($\bar{x}$) is an exponential function and therefore locally linear. The effects of small changes can thus be approximated as linear: $\bar{x}\ ( {c \cdot n,\ A} ) = \ c \cdot \bar{x}( {n,A} )$ for small changes: *c* ≈ 1. For example, a 5% decrease in the maximum number of targets will result in 5% decrease in bound targets. Therefore, it is not necessary to calculate the mean number of bound targets for each subpopulation with different molecular content. As a result, elimination rates can be more effectively calculated at a given antibiotic concentration *A*, if we already know one representative subpopulation $\bar{x}( {n,\ A} )$, where the heterogeneity in the population can be measured with the parameter *n* (for example, number of efflux pumps or number of targets).

(5) \begin{eqnarray*} {\delta ^{\rm{*}}} \left( {\bar{x}\left( {c \cdot n,{\rm{\ }}A} \right)} \right) = {\rm{\ }}{\delta ^{\rm{*}}}\left( {c \cdot \bar{x}\left( {n,{\rm{\ }}A} \right)} \right) \end{eqnarray*}

Finally, the time–kill curves of all subpopulations (e.g. with n target molecules, but more generally with a given phenotype *i*) have to be summed up to yield a time–kill curve of the entire bacterial population:

(6) \begin{eqnarray*} {B_{total}}\left( t \right) = \mathop \sum \limits_i {B_i}\left( 0 \right){e^{{\delta _i}t}} \end{eqnarray*}

In order to be able to compare the effects of persistence on treatment length (described below), we are assuming that the net growth rate for all subpopulations is negative. Otherwise the increase in treatment lengths will be infinite when some subpopulations have a positive net growth. This makes the comparison of different mechanisms difficult. However, as antibiotics are generally administered at multiples of the MIC this is often a reasonable assumption.

## RESULTS

**Figure 3.**
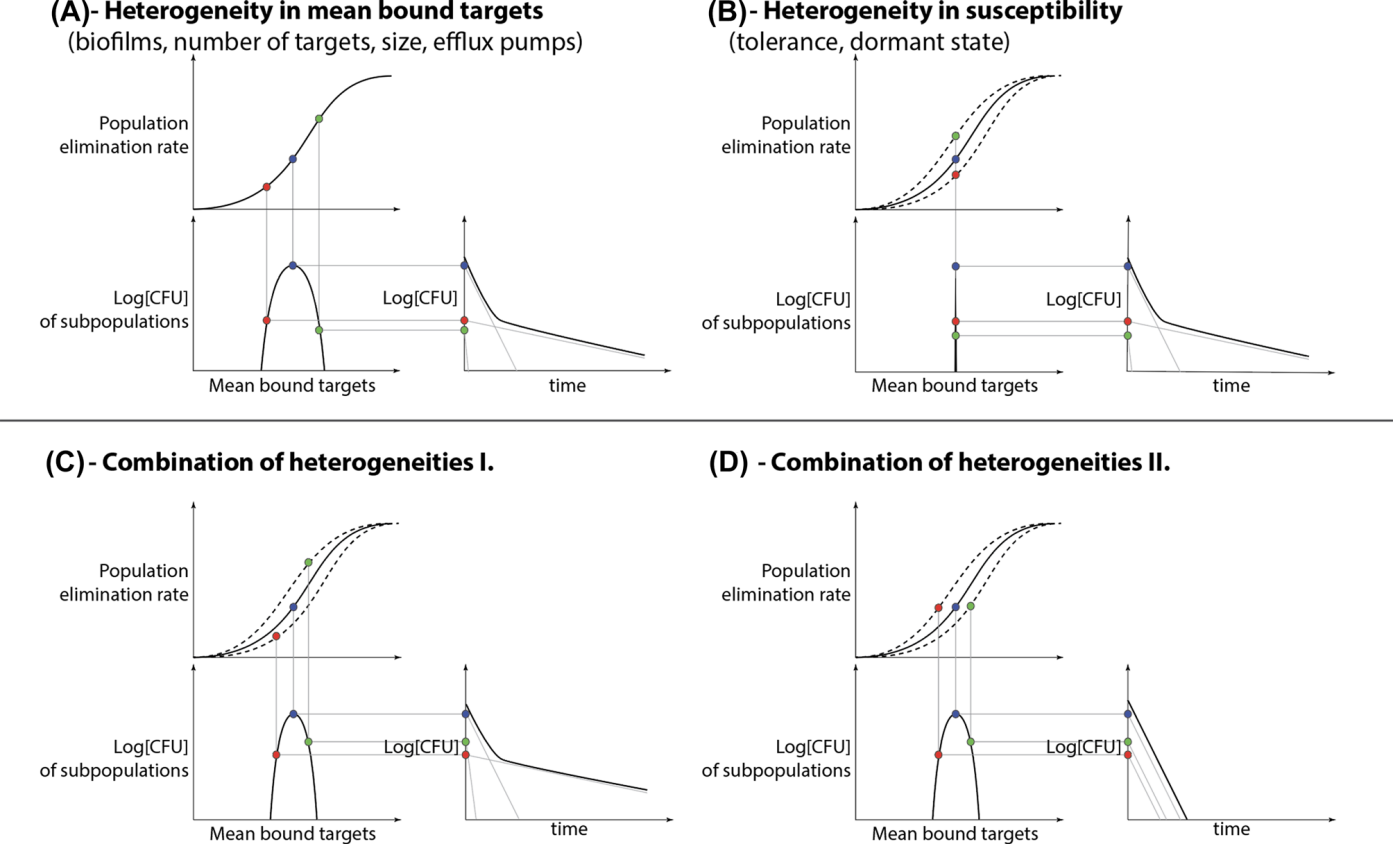
Bacterial populations with heterogeneities can show persistent behavior. These plots demonstrate how the model can be applied to various heterogeneities in the population. **A**, Shows the case where the assumed persistence mechanism causes a heterogeneity (only) in the number of bound targets. **B**, Where the assumed persistence mechanism causes a heterogeneity (only) in the susceptibility to the killing agent. **C** and **D**, Shows the combination of the two: whether heterogeneity results in persistent behavior depends on the relationship between the two heterogeneities within the population.

We first set out to investigate how different degrees of population heterogeneity in the number of target molecules affect persistence, defined as a slowdown in bacterial killing at a constant drug concentration. If we assume the same threshold (absolute number of bound targets, not percentages) for elimination, all subpopulations will have the same effective elimination curve. The heterogeneity in target molecules results, in a first approximation, in the same degree of heterogeneity in the number of bound targets for each subpopulation. This places the subpopulations at different points on the effective elimination rate curves. (see Fig. 3A). Therefore, small heterogeneities within the number of targets (1%–5% standard deviation around the mean) can lead to persistent behavior in the population of bacteria.

The same approach can be used for having different elimination rates while having the same number of mean bound targets as a different mechanism for persistence (see Fig. 3B). Finally, these two can be combined to account for more general cases. For example, when the subpopulations with different numbers of targets also differ from each other in the threshold of bound targets required to eliminate a single cell (i.e. different susceptibilities). The interplay between the change in susceptibility and bound targets can still cause persistent behavior, depending on their relationship (see Fig. 3C and D).

### Estimating treatment length

In this work, we have defined treatment length as the time point where all bacterial subpopulations are eliminated. We calculate the time to extinction for each individual subpopulation of bacteria and determine which is longest one. However, this necessitates the simplification that the eliminations of subpopulations are independent of each other: upon lysing the cells do not release their targets to the extracellular space. Furthermore, we neglect the decrease in extracellular antibiotic concentrations due to the uptake by cells. The goal of approximating the effects of heterogeneity and binding kinetics was to be able to reduce persistence models into two parts: the elimination rate as a function of a certain persistence mechanism and the distribution of subpopulations exhibiting various degrees of this persistence mechanism. This way, the treatment length for each subpopulation can be estimated by dividing the two functions (Equation 7). The resulting function shows the time points where the given subpopulations drop below one bacterium. This is best demonstrated by Fig. 1: as the Y-axis is logarithmic, exponential decays are straight lines that cross the X-axis at one individual bacterium. Therefore, (at constant antibiotic concentrations) dividing the logarithm of subpopulation size at t = 0 with their corresponding elimination rates will give us the time point the given subpopulation will go below one bacterium. We assume that at this time point they are eliminated, see Fig. 1.

(7) \begin{eqnarray*} {t_{\it survival}}\ \left( {\bar{x}} \right) = \frac{{{\rm{Log}}[{B_{total}}\left( {\bar{x}} \right)]}}{{{\delta ^*}\left( {\bar{x}} \right)}}\ , \end{eqnarray*}

where ${B_{total}}( {\bar{x}} )$ is the size of the subpopulation that has the mean of $\bar{x}$ bound targets (as depicted on the bottom left subplots on Fig. 3).

**Figure 4.**
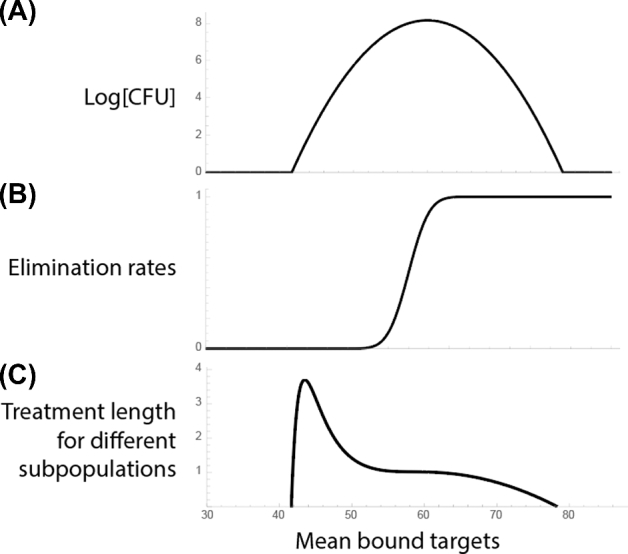
Estimation of treatment length. This plot demonstrates how treatment length for different subpopulations (**C**) can be estimated from the heterogeneity in the population (**A**) and the elimination rate function (**B**). **A**, Shows the heterogeneity in the mean bound targets within the population, here the majority of the population is simply the median of this distribution. **B**, It is the elimination rate function for the population, here we assume that the subpopulations only differ from each other in the mean number of bound targets. **C**, It is shows the assumed treatment length for each subpopulation (obtained with Equation 8), assuming that each of the subpopulations are independent of each other and the treatment length can be calculated as the time point where the given subpopulation goes below 1 bacterium.

If we normalize Equation (7) with time it takes to eliminate the largest subpopulation (the median of the population distribution, Fig. 4A), we get the fold-increase in time it takes to eliminate each subpopulation compared to the majority (see Fig. 4 and Equation 8).

(8) \begin{eqnarray*} {t^{\rm{*}}}{{\rm{\ }}_{\it survival}}\ \left( {\bar{x}} \right) = {t_{\it survival}}\left( {\bar{x}} \right)/{t_{\it survival}}\left( {{{\bar{x}}_{majority}}} \right) \end{eqnarray*}

**Figure 5.**
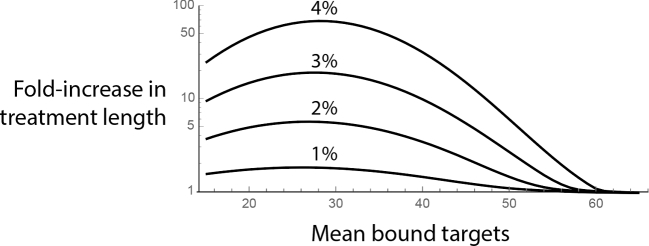
Increase in treatment length caused by persistence. This figure shows the fold-increases in treatment length (using Equation 8) for antibiotic concentrations that bind to 15%–70% of targets (threshold of elimination is 50 bound targets). The different curves are for different levels of heterogeneities in the number of maximum targets within the population. As the plot demonstrates, even small heterogeneities can cause a substantial increase in the required treatment length.

In Fig. 5, to demonstrate the estimation of treatment length and to reproduce the results of (Abel zur Wiesch *et al.*^2015^), we have plotted increase in treatment length for 1%, 2%, 3% and 4% heterogeneity in bound targets, meaning that the different subpopulations show a normal distribution in the number of (mean) bound targets, with the standard deviation of σ = 0.02$\ \bar{x}\ $(for a 2% heterogeneity). This can be due to the same heterogeneity in the number of targets among the subpopulations of bacteria as discussed above. Here, above a 2% heterogeneity, persistent bacteria take substantially longer to eliminate than the majority of the population. This is consistent with our results in (Abel zur Wiesch *et al.*^2015^), where we have shown that a 2% heterogeneity in bound targets is sufficient to show persistent behavior. The parameters used are *k_d_* = 10 ^− 5^, number of targets *n* = 100 (gyrase, (Malmström *et al.*^2009^; Maier *et al.*^2011^)); the cells are eliminated when more than 50 of their targets are bound (independently of the number of targets, n). The distribution around the mean (used in Equations 7 and 8, see Fig. 2C) was obtained by simulating the reaction kinetics using Gillespie simulations for the given *kd* and *n*. It is a normal distribution with the standard deviation of σ = 4.

### Using experimental data in our model

In this subsection, we demonstrate how to use an experimental dataset in our model. As the purpose of this subsection is to demonstrate a workflow, for the sake of brevity, we present a hypothetical and idealized scenario. We use the measurements of (Bergmiller *et al.*^2017^) which measured the distribution of growth rates in cells exposed to the bacteriostatic antibiotic tetracycline with single cell microscopy and thereby the heterogeneity in treatment response. They have also quantified a trait that contributes to this heterogeneity, the number of efflux pumps. This heterogeneity arises from biased partitioning during the replication of bacteria, which results in the daughter cells’ having fewer efflux pumps and therefore an efflux activity of 85–90% of the mother cells’ (measured dye uptake).

**Figure 6.**
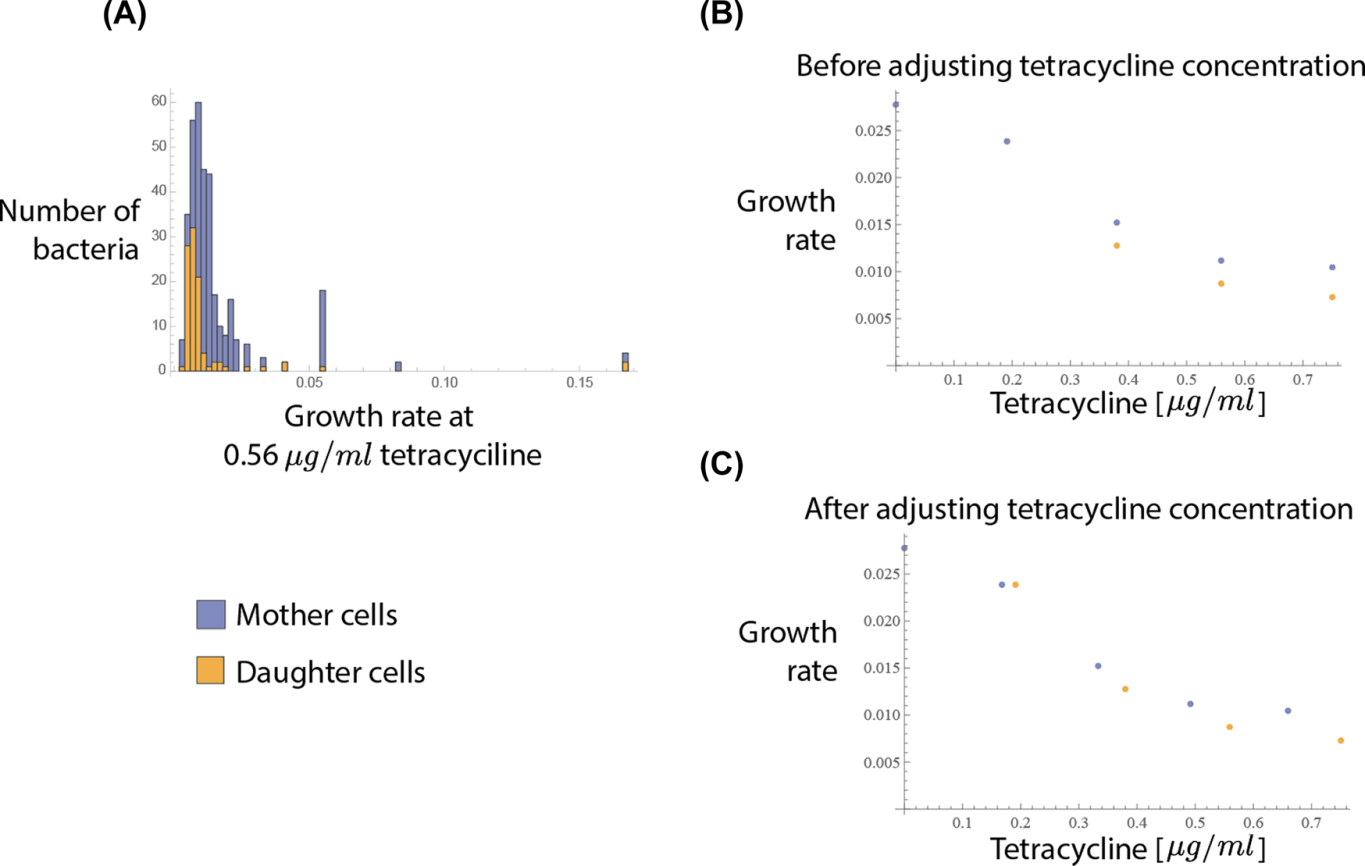
Using an experimental data set in the model. This plot illustrates the workflow of using an experimental dataset (taken from (Bergmiller *et al.*^2017^)) in the model. The dataset describes the change in replication rates under tetracycline exposure due the biased partitioning of efflux pumps between mother and daughter cells. **A**, Shows the histogram of measured growth rates at a specific antibiotic concentration for both the mother and daughter cells. **B**, Plots the medians of all the measured distributions at various antibiotic concentrations. In (**C**) the antibiotic concentrations for the mother cells have been adjusted to account for the increased efflux activity measured in the dataset that shows that the mother cells have 10% less dye taken up due to the higher numbers of efflux pumps. Here we have only used small part of the dataset, just to demonstrate the workflow from the experimental data to our model, in this case Fig. 3A.

To illustrate how to investigate such a dataset, we first obtain dose response curves for mother and daughter cells. Here, we assume that the only difference between the two generations is the number and activity of efflux pumps. Therefore, we take the median of both population growth rates to acquire a difference between the two generations (Fig. 6A and B). Next, we estimate the reduced (relative) drug concentrations affecting the mother cells by multiplying the external drug concentrations with the measured difference in efflux activity (dye uptake) between daughter and mother cells. While a proper analysis is outside the scope of this paper, in Fig. 6 we demonstrate that after the adjustment of drug concentrations the growth rate curves of the daughter and mother cells get close to each other and intersect.

**Figure 7.**
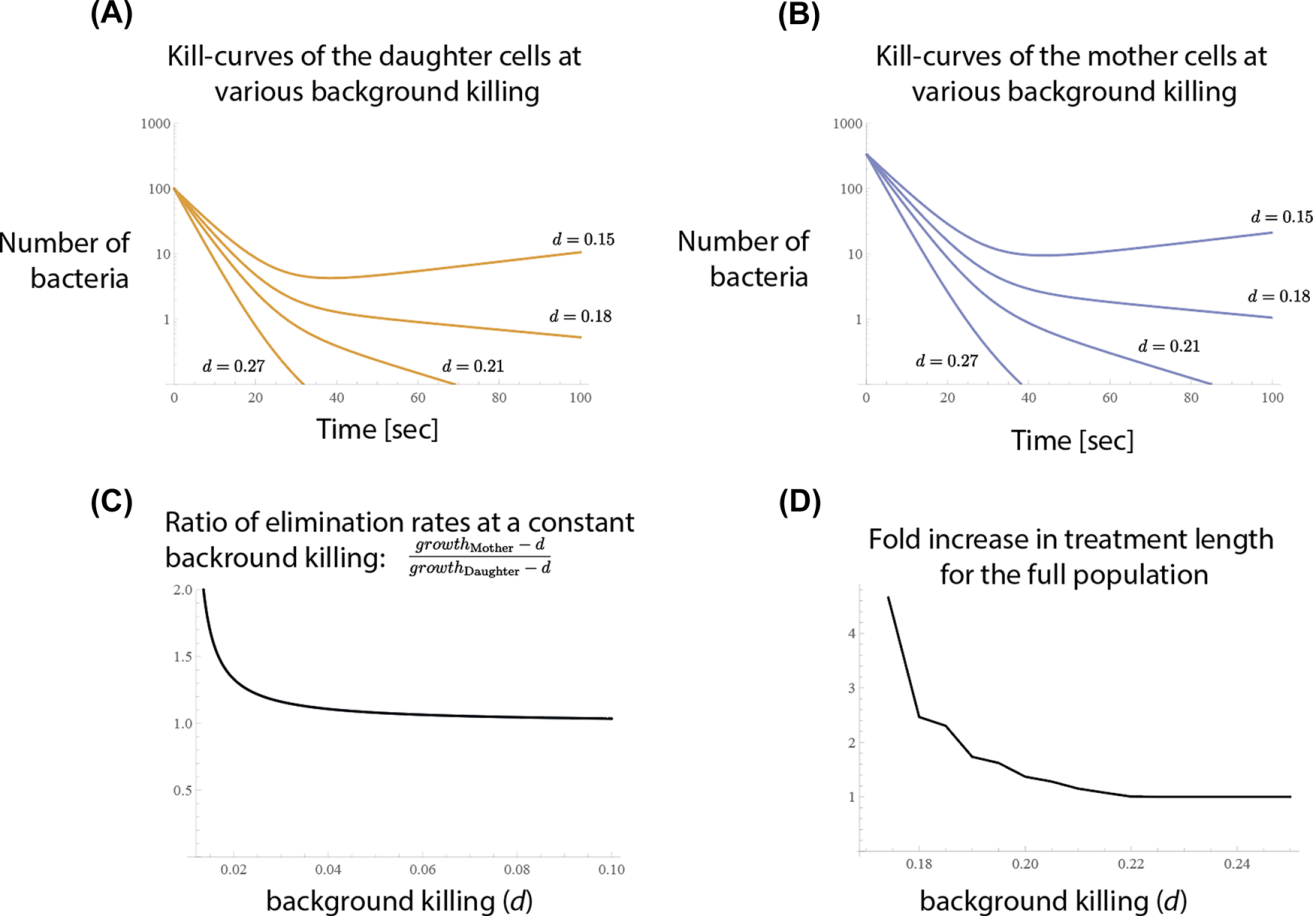
Separating persistence mechanisms with the model. This figure shows that the model can be used to separate different persistence mechanisms. To produce these plots, we have used the experimental data of (Bergmiller *et al.*^2017^). On (**A** and **B**) we have plotted the theoretical kill-curves based on the distribution of bacteria on Fig. 6A for mother and daughter cells respectively (using Equation 6). As the measurements were taken with a bacteriostatic drug (tetracycline), we have added a constant background killing to the population in order to create kill-curves from growth curves. It is important to note here that the heterogeneity that gives rise to the biphasic kill-curves are present in both the mother and daughter cells (see Fig. 6A). **C**, Shows the ratio of the median elimination rates for the mother and daughter cells. We have opted to show this as a measure of persistence instead of kill-curves as the kill-curves would also depend on the steady state population-sizes of the mother and daughter cells. This plot demonstrates we can expect biphasic kill-curves only at low background killing, provided that there are only two generations in a population. **D**, Shows the increase in time it takes to eliminate the whole population at different background killings due to the heterogeneity in growth rates. This curve is obtained similarly to Fig. 5 and Equation (8).

In this analysis, we can directly go to the step to estimate time–kill curves and the required treatment length because bacterial response to antibiotics (i.e. replication rate) was measured directly (Equations 5 and 6). The antibiotic used is tetracycline, a bacteriostatic antibiotic that mainly affects bacterial growth and is thought to act together with the immune system to clear infections. In order to create time–kill curves and calculate the time until the last bacterium would be eliminated, we generated a dataset by adding a constant elimination rate (caused by the immune system or an additional drug) to the available measured generation times. As a result, we get a distribution of eliminations rates for both mother and daughter cells. First, as within one generation we also have a distribution of growth (now elimination) rates (Fig. 6A), even mother or daughter cells in isolation should show biphasic kill-curves (Fig. 7A and B). This is possibly due to other persistence mechanisms as well as heterogeneity in efflux pumps within both the mother and daughter cell populations.

Next, in order to show the difference in the two generations, on Fig. 7C we have plotted the ratio of median elimination rates for the mother and daughter cells. We have opted to do this as the kill-curves would also depend on the steady state ratio of daughter and mother cells within a population (Figs. 3A and 4). This ratio or elimination rates can be used as a proxy to measure how biphasic a kill-curve is: the closer it gets to one, the smaller the difference is in the slopes for biphasic kill-curves. Fig. 7C demonstrates that if there is only one generation difference within the population, only low background killing rates (<0.02 [1/*s*]) would result in biphasic kill-curves. This is significantly different from Fig. 7A or B, where just for mother or daughter cells alone even at higher background killing (0.27 [1/*s*]) we saw biphasic kill-curve, indicating that only one generation difference in mother and daughter cells explains only a part of the heterogeneity in treatment response in this dataset. As shown in (Bergmiller *et al.*^2017^) natural bacterial populations have multiple generations present and consequently the mother cells in the experimental data might not have been from the same generation. Therefore, the differences in the number of efflux pumps might explain a larger part of heterogeneity in treatment response in this dataset, however investigating this is out of the scope of this work.

Taken together, Figs. 6 and 7 demonstrate how one can use our model with experimental data, separate mechanisms of persistence from others, and quickly estimate the parameter ranges where we would see biphasic curves with the given assumptions.

## DISCUSSION

The purpose of this work is to develop a mathematical model that can be used to estimate the increase in treatment length caused by persisters. At the same time, it should be general enough to encompass most proposed persistence mechanisms. We have demonstrated how the model presented in this paper can be used to describe persistence mechanisms that cause heterogeneities in either the susceptibility to the killing agent or the number of bound targets. Using a dataset from (Bergmiller *et al.*^2017^), we have demonstrated how our framework can be used to infer time–kill curves as well as the increase in necessary treatment length from measured heterogeneities in bacterial populations. We have also shown how this approach can help shedding light on the relative contributions of different persistence mechanisms.

Our mathematical framework can accommodate many different molecular mechanisms of bacterial persistence. These mechanisms can be separated into two different groups: they either affect the number of bound targets or the elimination rates. Examples for mechanisms that affect the number of bound targets are: (i) heterogeneity in the number of efflux pumps as it affects the concentration of antibiotics within the cells, but probably does not have a great impact on susceptibility to a given intracellular antibiotic concentration (Bergmiller *et al.*^2017^). (ii) Heterogeneity in cell volumes: if the relationship between cell volumes and number of targets is not linear, it will affect concentration of targets and therefore the number of bound targets (Rego, Audette and Rubin 2017). (iii) Biofilms: the antibiotic concentration is lower around bacteria that are deeper into the biofilms due to the imperfect penetration of antibiotics into biofilms, consequently the internal antibiotic concentrations in these cells will be lower as well (Lewis 2008). Examples of heterogeneities in how bacteria respond to a given number of bound targets include: (i) dormancy and (ii) increased lag time before replication if antibiotics only act on bacteria when they are actively replicating (Balaban *et al.*^2004^; Lewis 2007; Fridman *et al.*^2014^).

Moreover, as the model only describes heterogeneity in susceptibility to the killing agent and/or the number of bound targets subpopulation, the model can also be applied to immune-system mediated killing: AMPs, antibodies (Magnus and Regoes 2010), heterogeneity in the elimination of bacterial colonies by the immune system (Bumann 2015) or the elimination of cancer cells (Spencer *et al.*^2009^).

Our model is applicable when the bacterial population constantly declines, either because the antimicrobial concentrations are constantly above MIC or because the action of an antibiotic together with e.g. the immune system eliminates bacteria. Different types of models have to be employed in order to be able to investigate the effects of multiple-doses that are spaced far out or the effects of nonadherence. Furthermore, in our theoretical work we have only demonstrated how to work with unimodal distributions of persistence mechanisms. However, the application of our framework to skewed and not clearly unimodal experimental data demonstrates that it can be easily used to describe bi- or multi-modal distributions as well.

In summary, our approach allows the comparison and combination of multiple mechanisms within the same model that allows us to eliminate the inconsistencies when comparing different mechanisms, especially when it comes to fitting mathematical models describing different mechanisms of persistence to experimental or clinical data in different frameworks. Our work presents a simple and tractable way for estimating the effects of heterogeneities in bacterial populations, how they may result in persistent behavior, and how much they can increase the time until all subpopulations are eliminated. Ultimately, such models may aid decision making when it is safe to stop antibiotics without risking relapse due to remaining bacteria below detection limit.

## SUPPLEMENTARY DATA

Supplementary data are available at *FEMSPD* online.

Graphical Abstract Figure.This study shows a generalized mathematical framework applicable for most persistence mechanisms that can be used to estimate treatment length in persistent infections.